# Towards Efficient Early Warning: Pathobiology of African Swine Fever Virus “Belgium 2018/1” in Domestic Pigs of Different Age Classes

**DOI:** 10.3390/ani11092602

**Published:** 2021-09-04

**Authors:** Jutta Pikalo, Marie-Eve Schoder, Julia Sehl-Ewert, Angele Breithaupt, Ann Brigitte Cay, Coline Lhoëst, Willem van Campe, Laurent Mostin, Paul Deutschmann, Hanna Roszyk, Martin Beer, Sandra Blome, Marylène Tignon

**Affiliations:** 1 Friedrich-Loeffler-Institut, Suedufer 10, 17493 Greifswald, Germany; Jutta.Pikalo@fli.de (J.P.); Julia.Sehl@fli.de (J.S.-E.); Angele.Breithaupt@fli.de (A.B.); Paul.Deutschmann@fli.de (P.D.); Hanna.Roszyk@fli.de (H.R.); martin.beer@fli.de (M.B.); 2 Sciensano, Groeselenberg 99, 1180 Brussels, Belgium; Marie-Eve.Schoder@sciensano.be (M.-E.S.); annbrigitte.cay@sciensano.be (A.B.C.); Coline.Lhoest@sciensano.be (C.L.); Willem.VanCampe@sciensano.be (W.v.C.); Laurent.Mostin@sciensano.be (L.M.)

**Keywords:** African swine fever virus, Belgium, virulence, clinical course, domestic pigs

## Abstract

**Simple Summary:**

African swine fever (ASF) is a devastating viral disease of both wild boar and domestic pigs. Historically, the disease was mainly found in Sub-Saharan Africa. However, after the introduction of ASF into Georgia in 2007, the fatal disease spread to many European and Asian countries. In the absence of vaccines or treatment options, early detection of disease incursions is of paramount importance to limit the impact on animal health and pig industry. Thus, the biological characteristics of circulating virus strains must be known and communicated to practitioners and official veterinarians. Along these lines, the ASFV strain found in Belgium in 2018 was further characterized for its disease course in young and subadult domestic pigs. In general, clinical and pathological findings were in line with previous experiments utilizing highly virulent ASFV genotype II strains. However, in one of our experimental infections, four out of eight subadult domestic pigs showed milder signs and recovered, which was unexpected and points to an age dependency of clinical signs that could impact the early recognition of ASF incursions. We hope that communication of the available data will help practical and official veterinarians in the field to detect ASF as early as possible and thus minimize its impact.

**Abstract:**

African swine fever (ASF) is one of the most important and devastating viral diseases in wild boar and domestic pigs worldwide. In the absence of vaccines or treatment options, early clinical detection is crucial and requires a sound knowledge of disease characteristics. To provide practitioners and state veterinarians with detailed information, the objective of the present study was to characterize the ASF virus (ASFV) isolate “Belgium 2018/1” in subadult and weaning domestic pigs. To this end, two animal trials were performed. Trial A included eight subadult domestic pigs and trial B five weaner pigs. In general, clinical signs and pathological lesions were in line with previous studies utilizing highly virulent ASF genotype II viruses. However, in trial A, four subadult domestic pigs survived and recovered, pointing to an age-dependent outcome. The long-term fate of these survivors remains under discussion and would need further investigation.

## 1. Introduction

African swine fever (ASF) is a highly contagious and devastating disease of *Suidae*. The causative agent, African swine fever virus (ASFV), is a large double-stranded DNA virus, which belongs to the genus *Asfivirus* in the *Asfarviridae* family [1].

African swine fever has been endemic in many Sub-Saharan African countries and in Sardinia for many decades. However, after the introduction into Georgia in 2007, the disease spread to numerous eastern European countries and reached the European Union (EU) in 2014 with the first outbreaks in wild boar in the Baltic States and Poland [1,2]. In September 2018, the first case of ASF in wild boar was documented in Belgium [3]. The ASFV isolate associated with this outbreak, “Belgium 2018/1” [1,4], belongs to p72 genotype II and has shown high virulence in European wild boar [5]. Thus, it was comparable to other genotype II strains that are circulating in Europe and Asia, showing almost 100% lethality in animals of all age classes and sexes [6]. Clinical signs associated with such an infection include high fever, depression, inappetence, and respiratory distress [2,7]. Under experimental conditions, the animals showed the first clinical signs starting at 3–5 days post infection (dpi) [6], and animals developing an acute lethal disease course died within 7–13 dpi. Pathomorphological changes included enlarged, haemorrhagic lymph nodes; reddening of tonsils; congestion of spleen or splenomegaly; petechiae in different organs such as the kidney, colon, or urinary bladder; and lung and gall bladder wall edema [7]. Experimentally, the clinical course of ASF mostly has been studied in younger pigs (8–12 weeks), but there are some indications that the clinical course of ASF could be age-dependent under certain conditions [8].

In the absence of vaccines or other treatment options, early clinical detection is of paramount importance and requires detailed knowledge of clinical signs and pathological changes. Thus, biological strain characterization is important to inform farmers, practitioners, and state veterinarians involved in disease control [9].

Here, we report on the experimental inoculation of eight subadult domestic pigs and five weaner pigs for further characterization of the ASFV strain “Belgium 2018/1” and assessment of the influence of age on the clinical course and survival rate.

## 2. Materials and Methods

### 2.1. Experimental Design

The study comprised two animal experiments (trials A and B), that were performed to assess virulence and pathogenesis of genotype II ASFV from Belgium (ASFV strain “Belgium 2018/1”), and to collect suitable reference materials. Trial A was performed at Sciensano, Brussels, Belgium and trial B was carried out at the Friedrich-Loeffler-Institut (FLI), Greifswald-Insel Riems, Germany.

### 2.2. Animal Trials

#### 2.2.1. Trial A

The study comprised eight ASFV and ASFV antibody negative domestic pigs (Hypor hybrid × Pietrain breed) from a conventional farm weighing about 20 kg (10 weeks old). They were housed in a group at Sciensano in Animal Safety Level 3 facilities on slatted floors with water and food ad libitum for the duration of the experiment. The experiment was authorized by the Ethical Commission of Sciensano under N°20190614-01 and approved by the Biosafety commission. Upon arrival, the animals were randomly marked with ear tags starting from 1 to 8.

As these animals were part of another trial, each animal received an intramuscular injection of 1 mL of saline solution (mock injection) after one week acclimation. A second mock administration was performed in the same manner 23 days after the first injection.

At 49 days post mock injection and day 0 for this experiment, the subadult animals (18 weeks of age, weighing between 60 and 80 kg) were inoculated nasally using a small nebulizer (1-mm spray opening) fixed on a syringe to drip the infectious dose into the nostrils. Each animal received 4 mL virus suspension (2 mL per nostril) containing 1 × 10^4.3^ hemadsorbing units (HAU)/mL of ASFV “Belgium 2018/1”. The inoculum was cultivated on PBMC following standard procedures (EURL protocol: https://asf-referencelab.info/asf/images/ficherosasf/PROTOCOLOS-EN/SOP-ASF-VI-1REV2018.pdf (accessed on 28 August 2021). Following the initial protocol that had been drafted assuming acute disease courses, the experiment was terminated at 18 dpi. Due to the lack of high containment capacities and organizational reasons, the study could not be extended beyond 18 dpi.

Upon inoculation, body temperature and clinical parameters of all animals were assessed daily based on a harmonized scoring system as previously described [2]. Evaluated parameters are anorexia, recumbence, skin hemorrhage/cyanosis, swelling, breathing/coughing, ocular discharge, digestive trouble, and temperature. Based on the severity of the clinical signs, zero to three score points were awarded per parameter. The sum of points was recorded as the clinical score (CS). Temperatures higher than 40.5 °C were considered as severe fever, whereas temperatures higher than 39.7 °C (average +3 SD) were considered as mild fever. Animals reaching the humane endpoint of three subsequent days with severe fever (>40.5 °C), 9 score points, or were suffering unacceptably without reaching the endpoint score, were killed by electrocution and exsanguination.

Blood and serum samples were collected prior to inoculation, 3, 7, 10, 14, and 18 dpi at the day of euthanasia. Necropsy was performed on all animals, and at the same time, tissue samples (lymph nodes, spleen, tonsil, lung, liver, and kidney) and blood (EDTA, serum) were collected.

#### 2.2.2. Trial B

The study comprised five ASFV and ASFV antibody negative domestic weaner pigs (German Landrace × Large White) from a conventional farm weighing 20 to 25 kg. They were kept in the high containment facility (L3+) of the FLI. The animal experiment was approved by the competent authority (Landesamt für Landwirtschaft, Lebensmittlsicherheit und Fischerei (LALLF) Mecklenburg-Vorpommern, Rostock, Germany) under reference number LALLF 7221.3-2-011/19. Upon arrival, all animals were ear-tagged individually with numbers from 16 to 20. Over the course of the trial, the animals were fed a commercial pig feed with hay cob supplement and had access to water ad libitum. The animals were kept in one group and received species specific stable enrichment.

After an acclimatization phase, the animals were inoculated oro-nasally with 2 mL virus suspension containing 2 × 10^4.6^ HAU/mL ASFV “Belgium 2018/1”. The experiment was carried out until 9 days post-infection, when all animals had reached the humane endpoint as defined above. Upon inoculation, clinical scoring was performed as described for trial A. The same endpoint definitions were applied. Animals reaching the humane endpoint were euthanized through intracardial injection of embutramide (T61, Merck, Darmstadt, Germany) after deep sedation with tiletamine/zolazepam (Zoletil^®^, Virbac, Carros, France), ketamine (Ketamin 10%, Medistar, Ascheberg, Germany) and xylazine (Xylavet^®^ 20 mg/mL, CP-Pharma, Burgdorf, Germany) or ketamine (Ketamin 10%, Medistar, Ascheberg, Germany) and azaperone (Stresnil^TM^ 40 mg/mL, Elanco, Bad Homburg, Germany). Blood samples were collected prior to inoculation and at the day of euthanasia. At necropsy tissue samples (lymph nodes, spleen, tonsil, lung, liver, and kidney) and blood (EDTA and serum) were collected from all animals.

### 2.3. Virus Inoculum

#### 2.3.1. Trial A

The inoculum, ASFV “Belgium 2018/1”, was isolated by the Belgian national reference laboratory (NRL) for ASF at Sciensano from a wild boar carcass found in the Belgian municipality Etalle (Luxembourg region) [3]. The isolate belongs to genotype II and is closely related to strains circulating in eastern Europe [4] and beyond.

For the animal trial, cell culture supernatant was prepared on porcine peripheral blood monocytic cells (PBMCs) with a final titer of approximately 1 × 10^4.3^ HAU/mL. The titer was confirmed by an end-point back titration of the inoculum and calculated using the Reed and Muench method [10]. Titers were expressed as the amount of virus causing hemadsorption in 50% of infected cultures (HAU 50/mL).

#### 2.3.2. Trial B

The inoculum, ASFV “Belgium 2018/1”, was shipped from the Belgian NRL for ASF at Sciensano to the NRL for ASF in Germany at the FLI. For animal trial B, culture supernatant was prepared with a final titre of approximately 1 × 10^4.6^ HAU/mL. The titre was confirmed by an end-point back titration of the inoculum and calculated as described in trial A.

### 2.4. Cells for Virus Titration

#### 2.4.1. Trial A

All virus titrations and haemadsorption tests were carried out using PBMC-derived macrophages according to the protocol of the European Union Reference Laboratory for ASF in which harvesting of the PBMC by buffy coat had been replaced by separation on SepMate™ PBMC Isolation Tubes (STEMCELL Technologies, Vancouver, BC, Canada) with Ficoll-Paque™ PLUS Media (GE Healthcare, Chicago, IL, USA).

#### 2.4.2. Trial B

All virus titrations and hemadsorption tests were carried out using PBMC-derived macrophages. PBMCs were obtained and treated as previously described [11].

### 2.5. Pathology

#### 2.5.1. Trial A

Full autopsy was performed on all subadult domestic pigs infected with the ASFV strain “Belgium 2018/1”. Pigs were investigated macroscopically, and all lesions documented.

#### 2.5.2. Trial B

Full autopsy was performed on all domestic weaner pigs infected with the ASFV strain “Belgium 2018/1”. Pigs were evaluated macroscopically according to a scoring system published by Galindo-Cardiel et al. [12] with slight modifications [6].

### 2.6. Processing of Samples

#### 2.6.1. Trial A

Serum samples were obtained from native blood through centrifugation at 2.500× *g* for 20 min at 20 °C. Aliquots were stored at −80 °C until further use.

Tissue samples were collected during necropsy and stored at −80 °C. Fragments of about 100 mg tissue were homogenized in 1 mL phosphate-buffered saline (PBS) with 2 metal beads using a TissueLyser II (Qiagen^®^ GmbH, Hilden, Germany) for 2 × 2 min at 25 Hz before nucleic acid extraction.

#### 2.6.2. Trial B

Serum samples were obtained from native blood through centrifugation at 2.500× *g* for 20 min. Aliquots were stored at −80 °C until further use.

Tissue samples were cut into pea-sized fragments during necropsy and were stored at −80 °C for future use. One fragment was homogenized in 1 mL phosphate-buffered saline (PBS) with a metal bead using a TissueLyser II (Qiagen^®^ GmbH, Hilden, Germany) for 3 min at 30 Hz before virus isolations (haemadsorption tests) and qPCRs were performed.

### 2.7. Pathogen Detection—Nucleic Acid Extraction and Real-Time PCR

#### 2.7.1. Trial A

Detection of viral genome was done in blood, serum, and tissues using real-time PCR (qPCR). For qPCR, viral nucleic acids were extracted from blood and serum using the IndiMag Pathogen Kit (Indical Bioscience, Leipzig, Germany) on the Indimag48^®^ extraction platform (Indical Bioscience, Leipzig, Germany) and for tissue, the High Pure Viral Nucleic Acid Kit (Roche Applied Science, Penzberg, Germany) was used. All qPCRs were performed using the primers and probes for ASFV and endogenous gene beta-actin published by Tignon et al. [13] in AgPath-ID™ One-Step RT-PCR Master mix (Applied Biosystems, Foster City, USA) as described in Schoder et al. [14]. All PCRs were performed using a LC480^®^ cycler (Roche, Basel, Switzerland). Results of the qPCR were recorded as quantification cycle (Cq) values. Using a dilution series of an in-house ASFV DNA standard, the genome copies in the respective samples were determined. For generation of the ASFV standard, p72 gene from Lisbon60 strain (genotype 1) was amplified and cloned in pCR2.1 (Invitrogen, Carlsbad, CA, USA) for further multiplication in competent *E. coli*. The plasmid was extracted with Plasmid Plus Maxi Kit (Qiagen, Hilden, Germany) and linearized by restriction with BamHI. Subsequently, the DNA concentration was determined by spectrophotometry using a Nanodrop 2000 c (Thermo Fisher Scientific, Waltham, MA, USA) and the exact number of DNA molecules was calculated using an online tool (http://www.molbiol.edu.ru/eng/scripts/0107.html (accessed on 25 March 2020).

#### 2.7.2. Trial B

Prior to real-time PCR analysis, viral nucleic acids from all samples were extracted using the NucleoMag VET kit (Macherey-Nagel, Düren, Germany) on the automated KingFisher 96 flex platform (Thermo Fisher Scientific, Waltham, MA, USA) according to the manufacturer’s recommendations. Subsequently, nucleic acids were analyzed using the qPCR protocols published by King et al. [15] and Tignon et al. [13] on a Biorad CFX real-time cycler (Bio-Rad Laboratories, Hercules, CA, USA). For each qPCR, a quantification cycle (Cq) value was determined. Using a dilution series of the same standard as described in trial A, which was provided by Sciensano (Belgium), the genome copies in the respective samples were determined.

### 2.8. Antibody Detection

#### 2.8.1. Trial A

Sera were tested in three commercially available antibody ELISAs. In detail, ASFV p72-specific antibodies were detected using the INGEZIM PPA COMPAC ELISA (Ingenasa, Madrid, Spain), the ID Screen ASF competition ELISA (IDVet, Grabels, France) for antibodies against p32, and the ID Screen^®^ African Swine Fever Indirect (IDVet, Grabels, France) for antibodies against p32, p62 and p72. The tests were carried out according to the manufacturer’s instructions.

In addition, serum samples were tested in the indirect immunoperoxidase test according to the standard protocols provided by the European Union Reference Laboratory for ASF with slight modifications regarding the virus strain (Lisbon60 ASFV strain adapted on Vero cells). Over the study period, results were recorded in a qualitative way (positive/negative). Sera taken upon necropsy were end-point titrated in log2 steps to obtain semi-quantitative antibody titers.

#### 2.8.2. Trial B

Serum samples were tested as described for trial A. Slight modifications concerned the virus strain. Here, a cell culture adapted variant of genotype II ASFV “Armenia 2008” was used for the indirect immunoperoxidase test.

### 2.9. Statistical Analysis

Initial data recording and analyses (comparison of mean values, transformation of values) were done using Microsoft Excel 2010 (Microsoft Germany GmbH, Munich, Germany).

GraphPad Prism 8 (Graphpad Software Inc., San Diego, CA, USA) was used for graph creation.

## 3. Results

### 3.1. Clinical Findings

#### 3.1.1. Trial A

**Figure 1:**
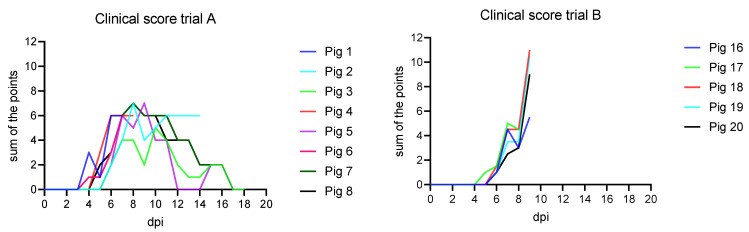
Clinical curve of trials A and B compared. In trial A, the maximum score of the animals was a sum of 7 points at 8 and 9 dpi compared to trial B, where animals reached scores of up to 11 points at 9 dpi.

Following nasal inoculation, all animals developed unspecific clinical signs starting from day 4 post inoculation (pi) (see Figure 1). The signs included general depression, lack of appetite and unwillingness to stand up, reduced mobility, tremor, reddened skin, cyanosis on the ears and snout, hunched-up back, and respiratory distress. The highest score was reached at 8 and 9 dpi with a maximum of 7 points.

**Figure 2:**
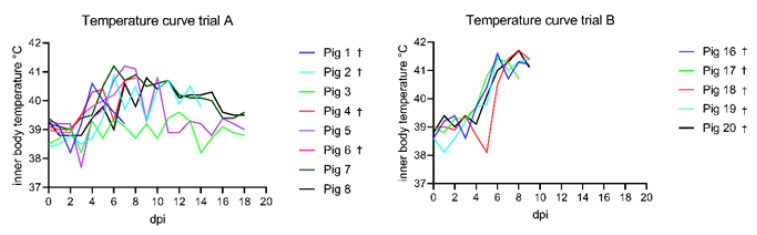
Temperature curves of trials A and B compared. In general, subadult animals of trial A showed lower temperatures compared to the weaner group of Trial B. The maximum temperature recorded in trial A was 41.2 °C at 7 dpi. In trial B, the highest temperature was 41.7 °C at 8 dpi. Animals that showed an acute lethal disease course and reached the humane end point (or died) are marked with a cross.

The onset of fever was observed as early as 4 dpi and the climax of the illness was observed around 8–9 dpi (see Figure 2). The peak of fever was observed at 7 and 8 dpi with the highest number of severe feverish animals. Afterwards the intensity of the fever decreased but remained present in a mild form until 14 dpi. No temperature increase was observed during the infection period for animal #3 despite the presence of other clinical signs. The average number of days with fever was as follows: for severe fever 2.75 +/− 2.25 and for mild fever 5.5 +/− 3.85.

Ethical euthanasia was performed between 7 and 14 dpi on animals with clinical scores between 6 and 7 and showing persistent severe fever (3 subsequent days) and/or very poor reactivity. Starting from 12 dpi, the remaining animals started to recover. At the end of the experiment (18 dpi), the surviving pigs, four out of eight, presented no more clinical signs.

#### 3.1.2. Trial B

Following oronasal inoculation, all animals developed severe, unspecific clinical signs starting from day five pi (see Figure 1). The signs included general depression, lack of appetite and mobility, hunched-up back, ataxia, and respiratory distress. The onset of fever was observed at 5 dpi (see Figure 2). On 9 dpi, all animals reached the humane endpoint except one (pig 17), which died overnight. The clinical scores ranged from 5.5 up to 11.

**Figure 3:**
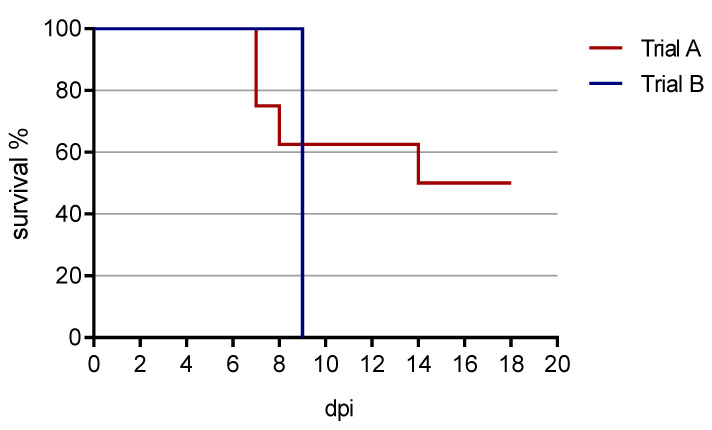
Survival curve from both animal trials. In trial A, four out of eight pigs survived until the end of the experiment at 18 dpi. In trial B, all animals were euthanized by 9 dpi (reaching the humane endpoint). One animal died spontaneously in the night from 8 to 9 dpi.

The survival curve from both animal trials is presented in Figure 3.

### 3.2. Pathomorphological Findings

#### 3.2.1. Trial A

Animals were euthanized either during the experiment for ethical reasons (severe fever for more than 3 consecutive days or high clinical score or poor health condition) or at the end of the experiment (18 dpi). At necropsy, various pathological patterns were observed: from asymptomatic to typical ASF lesions of varying severity: generalized hemorrhagic lymphadenopathy especially of the gastrohepatic lymph nodes, congestion of the spleen, and multiple hemorrhages in several organs, particularly in the kidneys (Table 1).

#### 3.2.2. Trial B

Five ASFV “Belgium 2018/1” infected domestic pigs reached the humane endpoint at 9 dpi (n = 4) or died spontaneously (n = 1) and were submitted to necropsy. At gross pathologic investigation, all infected pigs revealed typical lesions indicative of ASF. All pigs showed severely hemorrhagic enlarged lymph nodes with the gastrohepatic and renal lymph nodes mainly affected. Renal petechiae were present in all pigs and were mainly confined to the renal cortex and to a lesser extent to the medulla. Four out of five pigs had ascites. Marked sanguineous effusion was present in two pigs, while the other two showed accumulation of serous fluid. Likewise, rather mild, serous to sanguineous pleural effusion was present in three animals. Bruises of variable size appeared in four pigs. In individual cases, mild to moderate perirenal and gall bladder wall edema, mucosal petechiae in the urinary bladder, bilateral cyanosis of the ears, mild multifocal pulmonary consolidation, and alveolar edema were observed (Table 2).

### 3.3. Pathogen Detection

#### 3.3.1. Trial A

Prior to infection, all animals were tested negative for ASFV nucleic acids by qPCR in blood samples. After infection, the presence of the virus was first detected in blood samples of 3 animals collected at 3 dpi. All remaining animals were detected ASFV PCR positive at the following sampling time point (7 dpi), except one animal (#3) which became positive 10 dpi. Once detected positive, the animals remained positive until death by ethical euthanasia or at the end of the experiment (18 dpi).

When detected at 3 dpi, the virus load was low (<25 copies/run). However, at 7 dpi and until 14 dpi, the virus load in blood was at a maximum with 1.29 × 10^3^ to 6.80 × 10^4^ copies/reaction (Table 3).

At the end of the trial, viral genome was detected in blood, serum, and all tissue samples (spleen, lung, lymph nodes, tonsil, kidney, and liver). Highest loads of viral genomes (>1 × 10^3^ to 1 × 10^4^ copies/reaction) were found in blood, serum, and tissues from animals presenting severe clinical signs, whereas low viral genome loads (<500 copies/reaction) were detected in the blood of animals that survived the infection (Table 4).

#### 3.3.2. Trial B

Prior to inoculation, all animals were tested negative for ASFV, ASFV antigen, and viral nucleic acids.

At the end of the trial, viral genome was detected in blood and all tissue samples (spleen, lung, lymph nodes, tonsil, kidney, and liver). Highest loads of viral genome were found especially in blood, serum, and spleen (Table 5).

### 3.4. Antibody Detection

#### 3.4.1. Trial A

Prior to inoculation, all animals were tested negative for ASFV antibodies (Ab) by ELISA tests.

**Figure 4:**
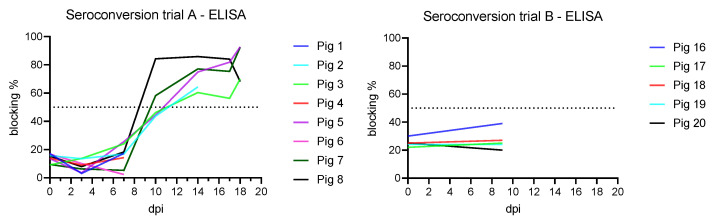
Seroconversion of the subadult animals from trial A detected on 10 dpi with INGEZIM PPA COMPAC ELISA (Ingenasa, Madrid, Spain). Weaner pigs from trial B showed no ELISA detectible seroconversion at 9 dpi. The ELISA cutoff is 50% blocking which is indicated as a dotted line in both graphs.

After infection, seroconversion against ASFV p72 was demonstrated using the INGEZIM PPA COMPAC ELISA (Ingenasa, Madrid, Spain) in the five still remaining animals at 10 dpi (two positive and three doubtful results) (Figure 4). With the ID Screen ASF Competition ELISA (IDVet, Grabels, France), which detects p32, all remaining animals were positive from 10 dpi, except one which already showed a questionable result at 7 dpi. By testing with the ID Screen ASF Indirect ELISA (IDVet, Grabels, France), three animals showed a questionable result at 10 dpi, one was negative, and one showed a positive antibody result from 10 dpi onwards. From 14 dpi and onwards, all four remaining animals were seropositive in all three ELISA assays.

With the confirmatory indirect immunoperoxidase test, seroconversion was observed starting from 7 dpi (qualitative result). At the end of the experiment (18 dpi), the surviving animals presented antibody titers between 2560 and 5120 (semi-quantitative result).

#### 3.4.2. Trial B

No antibodies were detected in the sera prior to inoculation or at the end of the trial on 9 dpi with all ELISAs (Figure 4). With the indirect immunoperoxidase test, however, the animals showed semi-quantitative antibody titers between 160 and 620 at 9 dpi.

## 4. Discussion

African swine fever virus has entered the European Union and many countries in eastern Europe and Asia, threatening animal health and agriculture alike. In the absence of a licensed vaccine or other effective treatment options, prevention through farm biosafety and the earliest possible detection of outbreaks are of utmost importance. Early detection is only assured if clinical signs are recognized promptly and interpreted correctly by farmers and practitioners. Thus, well-trained livestock farmers and veterinarians in practice and subsequently the competent authorities play a crucial role here. Basic data to inform these key persons can be obtained from animal experimental work, such as that carried out in the National Reference Laboratories (NRLs) as part of their tasks. These studies are not infrequently conducted to generate and archive relevant and well-characterized sample materials for validation and harmonization of diagnostic methods at the national level (Regulation (EU) 2017/625, Article 101), but provide space to map issues of pathogenesis and virulence of locally relevant viral variants. In this context, the present study was conducted in close cooperation with the Belgian and the German NRLs with the aim to further characterize the ASF virus isolate “Belgium 2018/1” in domestic pigs. Thereby, the focus was laid on the clinical courses and pathomorphological changes in different age classes of animals. While various studies with genotype II strains have been conducted in weaner pigs, studies with older animals are scarce. Although there are indications that no age dependency is involved in highly virulent strains [16], different courses have been described for moderately virulent strains [8]. In the study published by Post et al. [8], age had a marked effect on disease outcome, while the inoculation dose was secondary. The latter is consistent with studies describing similar, often lethal, courses with different doses of highly virulent virus strains [11].

In our combined study, inoculation of weaner pigs did not hold any surprise. After an incubation period of five days, which is in line with previous findings [17,18], all young animals developed an acute lethal disease course with high fever, general depression, anorexia, ataxia, and respiratory distress. All animals reached the humane endpoint or had died acutely by 9 dpi. It is noteworthy that again the clinical signs were severe but rather unspecific, leading to many differential diagnoses that could be relevant in the field. Necropsy findings were in line with previous studies using ASFV “Armenia 2008” [6]. Viral genome was found in organs, blood, and serum of all animals irrespective of the disease course. In line with previous studies, blood, spleen, and liver showed the highest copy numbers in the majority of animals. However, genome loads reflected the clinical course and timepoint of sampling. While viral genome loads in spleen were roughly 10,000 genome copies per run for animals sampled between days 7 and 9, less than 10 genome copies were found in recovering pigs at 18 dpi.

The inoculation of the older pigs took a different, rather unexpected turn. Although the Belgian virus hardly differs at the sequence level from the highly virulent strains from Armenia and Georgia [4], which showed rather age-independent courses in previous studies [16], only a 50% lethality was recorded in the present study until the end of the experiment at 18 dpi. Clinical signs were milder and pathomorphological findings reflected the clinical course (severe to absent lesions). It must be mentioned, however, that the capacity-related observation period of 18 days limits a reliable and complete statement on the final clinical outcome. Previous studies have shown intermittent and recurring viremia after 18 dpi [2]. In addition, it cannot be excluded from the clinical scoring and virus detection that at least one animal (#3) got infected only by contact (not following inoculation) and thus was slightly delayed. Nevertheless, the lack of findings in the pathological-anatomical examinations and low genome loads in organs indicate that the remaining animals, including animal #3, were true survivors or showed only a sub-clinical infection due to the lack of lesions. Survivors were rarely seen when using highly virulent ASFV strains of genotype II; however, they were reported. As an example, Gallardo et al. [2] reported on the survival of one out of eight pigs inoculated intramuscularly with the Lithuanian ASFV strain “LT14/1490”. The respective pig showed weak and intermittent peaks of viremia, and viral DNA could be detected in tissues at 38 dpi. However, no seroconversion was observed, which was different in our study where all survivors seroconverted. Animals surviving the acute phase were also reported by Walczak et al. [9]. In this study, two animals inoculated with the Polish ASFV “Pol18_28298_O111” strain (pig one got 1000 HAU and pig two got 500 HAU) developed chronic disease courses after a delayed incubation period (pig one 12 dpi: clearly visible clinical signs, like joint swelling and minor breathing disorders, moderate fever, and constant low virus load value in blood but without pathological lesions. Pig two 20 dpi: showed only moderate fever and enlarged submandibular lymph nodes) but had to be euthanized 24 and 32 dpi, respectively. While it is obvious that animals may survive, the long-term fate and epidemiological role of these survivors are still discussed controversially and need further long-term studies to allow final conclusions. While survivors may eventually recover completely, longer term virus excretion will impact on transmission dynamics. In a previous long-term study with a moderately virulent genotype I strain, virus was isolated from recovering pigs up to day 63 post infection [19].

Apart from age, application route and dose, the general health and immune status, genetic background (hybrid breed), and concomitant infections could impact on the clinical outcome and thus explain our observations. Indeed, due to the size of the sub-adult pigs, inoculation had to happen in standing position rather than in dorsal recumbency, which was used for the weaner pigs. This could have influenced the amount of virus that reached the tonsils. Adding to that, the dose is reduced when compared to body weight. However, as mentioned earlier, several studies showed severe courses upon low-dose infection and did not report a significant impact of the viral dose on the final outcome [2,9,11]. Whether an even lower dose would have led to more survivors or just less infected pigs could be debated. Considering the study reported by Pietschmann et al. [11], we could rather expect lower infection rates. The route itself can also play a role in the efficiency of infection. While trial A was conducted with nasal inoculation, trial B was done with oro-nasal inoculation. In this context, Howey et al. [20] reported on the variable efficiency of intranasopharyngeal (INP) and intraoropharyngeal (IOP) inoculations, especially using lower doses (10^2^ HAU). In this study, oropharyngeal infection was less efficient. However, infection was confirmed in all animals of our study upon either nasal or oral infection. Given that the full range of clinical outcomes and swift seroconversion in survivors were seen, a dose-related impact cannot be excluded but does not seem likely. Considering that both studies employed clinically healthy, commercial pigs, the impact of the general health status seems rather small.

With regard to the genetic background, differences in susceptibility are seen rather frequently in indigenous pig breeds in Africa [21,22]. Yet, our study involved only widely used pig breeds of Europe that may not differ markedly in their susceptibility, even if a different hybrid breed was used in trial A than in trial B. As genotyping of pigs was not carried out, no final conclusion on this aspect can be drawn.

Recently, the gut microbiota was discussed as an important factor for ASF susceptibility. Interestingly, fecal microbiota transplantation from warthogs to domestic pigs resulted in higher resistance of the latter [23]. While we cannot rule out such factors, we do not assume a high impact of the gut microbiota in our study.

The variability of clinical signs and outcomes in our study points to both a certain age-dependency and biological variability that has to be taken into account when communicating clinical signs and typical disease outcomes.

## 5. Conclusions

Taken together, we saw a variable clinical picture when considering all age classes of animals that could cause problems in the clinical evaluation of ASF under field conditions and in early warning scenarios. The final fate of the here observed survivors could not be addressed under the limited time scheme of our study and should be part of further long-term studies with older pigs.

The outcome of our study highlights the need for swift and reliable laboratory diagnosis, even when only mild to moderate clinical signs are detected. Easy and fast genome detection by qPCR did not pose any problems even with the surviving animals.

We hope that communication of the available data will help practical and official veterinarians in the field to detect ASF as early as possible and thus minimize its impact. The study outcome clearly underlines once again that clinical courses can be highly variable and non-specific. For this reason, exclusion diagnostics of ASF by sensitive qPCR methods should be routine, especially in light of the current situation with ASF outbreaks in several countries including Germany.

## Figures and Tables

**Table 1:** Overview of pathological findings per animal from trial A.

Animal	Day of Euthanasia	Cause	Major Pathological Observations
1	7 dpi	humane endpoint	▪ Hemorrhagic lymph nodes
2	14 dpi	humane endpoint	▪ Hemorrhagic lymph nodes ▪ Renal petechiae ▪ Congestion of spleen
3	18 dpi	end of experiment	▪ No evident lesion
4	8 dpi	humane endpoint	▪ Hemorrhagic lymph nodes ▪ Renal petechiae ▪ Congestion of spleen
5	18 dpi	end of experiment	▪ No evident lesion
6	7 dpi	humane endpoint	▪ Hemorrhagic lymph nodes ▪ Renal petechiae ▪ Congestion of spleen
7	18 dpi	end of experiment	▪ No evident lesion
8	18 dpi	end of experiment	▪ No evident lesion

**Table 2:** Overview of pathological findings per animal from trial B.

Animal	Day of Euthanasia	Cause	Major Pathological Observations
16	9 dpi	humane endpoint	▪ Multiple bruises ▪ Pulmonary consolidation ▪ Hydroperitoneum ▪ Renal petechiae ▪ Enlarged, hemorrhagic lymph nodes
17	9 dpi	died acutely over night	▪ Single bruise ▪ Subcutaneous hematoma on chest ▪ Pulmonary alveolar edema ▪ Renal petechiae ▪ Enlarged, hemorrhagic lymph nodes
18	9 dpi	humane endpoint	▪ Cyanosis of ears ▪ Hydrothorax ▪ Hydroperitoneum ▪ Pulmonary consolidation ▪ Renal petechiae ▪ Mucosal petechiae in urinary bladder ▪ Enlarged, hemorrhagic lymph nodes
19	9 dpi	humane endpoint	▪ Cyanosis of ears ▪ Multifocal bruises ▪ Hemothorax ▪ Hemoperitoneum ▪ Perirenal edema ▪ Renal petechiae ▪ Enlarged, hemorrhagic lymph nodes
20	9 dpi	humane endpoint	▪ Single bruise ▪ Hydrothorax ▪ Pulmonary consolidation ▪ Hemoperitoneum ▪ Gall bladder wall edema ▪ Renal petechiae ▪ Enlarged, hemorrhagic lymph nodes

**Table 3:** Trial A: real-time PCR results of blood from 0 to 18 dpi. Genome detection in blood is presented as genome copies per reaction (5 µL). nd: not detected; NA: due to technical and organizational problems samples could not be taken and analyzed. The color intensity indicates the relative level of viral loads (darker colors indicating higher loads, from light yellow to deep red).

Animal	0 dpi	3 dpi	7 dpi	10 dpi	14 dpi	18 dpi
1	nd	0.3	29,200			
2	nd	nd	1270	NA	4260	
3	nd	nd	nd	19	15	8
4	nd	0.7	15,100			
5	nd	nd	202	1290	353	284
6	nd	22	68,000			
7	nd	nd	401	1660	525	127
8	nd	nd	2810	2810	870	20

**Table 4:** Trial A: real-time PCR results of blood, serum, and organ samples at the day of euthanasia. Genome detection in blood, serum, and organs are presented as genome copies per reaction (5 µL). The color intensity indicates the relative level of viral loads (darker colors indicating higher loads, from light yellow to deep red). nd: not detected.

Animal	Day of Euthanasia	Blood	Serum	Spleen	Tonsil	Lymph Node	Lung	Liver	Kidney
1	7 dpi	29,200	1140	37,100	21,000	33,300	20,100	13,200	734
2	14 dpi	4260	595	4520	855	6600	820	2630	120
3	18 dpi	8	nd	4	57	43	479	2	1
4	8 dpi	15,100	1000	18,700	13,100	8760	9540	30,600	894
5	18 dpi	248	0.2	5	514	132	5	nd	2
6	7 dpi	68,000	15,200	43,000	56,700	37,000	59,600	224,000	13,800
7	18 dpi	127	0.1	3	423	319	8	2	1
8	18 dpi	20	0.02	10	102	7	6	4	3

**Table 5:** Trial B: real-time PCR results of blood, serum and organ samples at the day of euthanasia. Genome detection in blood, serum, and organs are presented as genome copies per run (5 µL). The color depth indicates the level of the viral load. From white to red indicates from low to high viral load.

Animal	Day of Euthanasia	Blood	Serum	Spleen	Tonsil	Lymph Node	Lung	Liver	Kidney
16	9 dpi	10,200	2320	4510	164	42	122	359	49
17	9 dpi	66,400	15,800	15,400	8560	9770	4520	48,600	3780
18	9 dpi	103,000	22,500	14,000	1900	589	5810	22,000	707
19	9 dpi	61,900	13,900	4610	3360	587	552	4040	500
20	9 dpi	78,000	27,100	23,000	18,900	3120	5680	11,000	987

## Data Availability

The data that support the findings of this study are available from the corresponding author upon reasonable request.
